# Frequencies and ethnic distribution of ABO and RhD blood groups in the Volta region of Ghana, towards effective blood bank services

**DOI:** 10.4314/ahs.v22i1.74

**Published:** 2022-03

**Authors:** George N Doku, William K Agbozo, Rabia A Annor, Priscilla E Mawudzro, Elizabeth E Agbeli

**Affiliations:** 1 Department of Physician Assistantship, School of Medicine and Health Sciences, Central University, Ghana

**Keywords:** ABO and RhD blood groups, Volta region of Ghana, towards effective blood bank services

## Abstract

**Background:**

Blood is an essential body fluid for the transport of substances to all parts of the body. Knowledge of blood group distribution within any population is important in determining the direction of blood bank inventory for emergency blood services.

**Objective:**

We report for the first time the blood group distribution pattern for the Volta region of Ghana.

**Method:**

Data were extracted and analyzed from 14,360 medical records of blood donors and recipients at seven major hospitals within the Volta region for a period of seven years (2012 to 2018)

**Results:**

ABO distribution within the region was 46.3%, 18.9%, 24.4%, 3.1%, 4.4%, 1.7%, 1.3% and 0.1% for O+, A+, B+, AB+, O-, A-, B- and AB- blood groups respectively. Rh (D)+ to Rh (D)- ratio was 92.5/7.5% respectively. Blood group O+ (>35 %) was highest in all ethnic groups in the region.

**Conclusion:**

Healthcare facilities in the region should adopt a strategy to stock-pile sufficient O+ blood which is the prevalent blood group in the region. All types of blood groups were reported hence our findings should provide information to guide clinical practice and/or blood transfusion services in the region.

## Introduction

Blood is an essential body fluid for the transport of substances such as nutrients, oxygen, hormones and enzymes to all parts of the body. Historically, the safety of blood transfusion services has relied greatly on the discovery of the different blood group systems^1^. Red blood cells have surface proteins, carbohydrates, glycoproteins and glycolipids, referred to as antigens, whose presence or absence serve as the basis for defining or characterizing blood group systems^2^.

Among the many human blood group systems known, the two most widely accepted for safe transfusion services are the ABO and Rhesus blood (Rh (D)) systems. The presence or absence of two antigens (A and B) gives rise to four blood group categories (A, AB, B, and O) of the ABO system. Similarly, Rh-positive and Rh-negative are two categories of the Rh(D) system, based on the presence or absence of Rhesus D antigen on the red cell surface^3^. According to ABO blood group/Rhesus factor nomenclature, a person can belong to either of the following eight blood groups: A Rh+ (A+), A Rh- (A-), B Rh+ (B+), B Rh- (B-), AB Rh+ (AB+), AB Rh- (AB-), O Rh+ (O+) and O Rh- (O-)^4^,^5^

Although the ABO and Rhesus groups are the same for all humans, factors such as geographical, ethnic, racial and tribal variations result in long-lasting differences in the frequency distribution of the blood groups^1^. Besides the above long-lasting differences, temporal changes in the frequency distribution may also occur due to socioeconomic developments that bring new people into the population as well as the tendency to move out to other settlements^6^. It is thus important to obtain established data on the frequency distribution for any notable population such as an ethnic group, a region or a state or a province in a country, etc., for effective blood transfusion services. Also, knowledge of a population's blood group distribution is reported to be useful in promoting health and general wellbeing of the population^7^,^8^,^9^.

In Ghana, there is paucity in the literature on the blood group distribution for each administrative region of the country as well as the nation as a whole. Meanwhile, this information is important and needed to guide the recruitment of blood donors in each region for effective blood transfusion services. The interest of our research team is, therefore, to address this paucity in the literature by investigating and reporting the ABO/Rh(D) blood group distribution in all administrative regions of the country and finally come out with a national database. Previous studies have reported the blood group distribution for Greater Accra^5^ and Eastern^10^ regions of Ghana, with other regions currently being investigated. This paper reports on the ABO/Rh (D) distribution pattern for the Volta region of Ghana.

## Methods

### Study setting

This study was done for the Volta region of Ghana, which represent one of Ghana's sixteen (16) administrative regions. The Volta region is at the eastern cardinal direction of the country and located west of Republic of Togo and to the east of Lake Volta (Figure 1). Ghana's administrative regions are co-inhabited by persons belonging to smaller ethnic groups. Ghana's many smaller ethnic groups can be placed under four broad ethnic groupings - Akans, Ewes, Ga-Adamgbes and Northerners. The Akans include the Fantis, Ashantes, Kwahus, Akwapims and many more. The Ewes include the Anlos, Avenors and many more. The Ga-Adamgbes comprise the Ga, Krobos, Ada and many more. The Northerners comprise the Hausas, Frafras, Dagombas and many more. All other subjects of non-indigenous Ghanaian family routes were classified as non-Ghanaian. The Ewe ethnic group is the native and largest ethnic group (68.5 percent of the population) in the Volta region.

### Study Design

A descriptive cross-sectional design with retrospective approach was used to retrieve data on the ABO/Rh blood group status of blood donors and recipients who visited the major health facilities within the region. Blood donations were from voluntary unpaid blood donor's and family replacement donors.

### Data source

Major hospitals situated across the entire region that provides blood transfusion services for the region's population (namely; Ho Regional Hospital in Ho, Comboni Catholic Hospital in Sogakope, the Sogakope District Hospital in Sogakope, St. Anthony Catholic Hospital in Dzodze, the Keta Municipal Hospital in Keta, the Sacred Heart Mission Hospital at Abor and the Margaret Maquart Hospital in Kpando) were selected. All medical records of blood donors and recipients available within a period of seven years (2012 to 2018) that the study was carried out were used. A total of 14,360 (7467 males and 6893 females) records were retrieved. A standardized extraction form was designed based on literature review5,10 and used to extract data including age, sex, ethnicity, blood group, rhesus status, etc. Data extraction was done by physician assistants who were recruited from the health facilities and trained by the authors on the data extraction techniques as research assistants.

### Laboratory test

Blood services within the region are generally done in these main hospitals.

ABO blood typing in donors and recipients were carried out by trained medical laboratory scientist using the model standard operating procedures for blood transfusion service by the W.H.O. Suspension of participants red cell specimen/span> was reacted with antisera (anti-A and anti-B) regents. Positive agglutination indicated the presence of corresponding antigen (agglutinogen) on the red cells. Rh typing was similarly carried out by adding antibody-D (anti-D) serum to the participants blood sample. The appearance of clumping (agglutination) indicated Rhesus D antigen present (positive).

### Statistical analysis

The data was first recorded in Microsoft Excel for Windows 10 software. Authors ensured that data entered was delimited and lined-up in proper columns, no missing values for key parameters and scan for anomalous values, performed statistical summaries, all as quality control check for data entered. Analysis was done using SPSS statistical software (Version 20; Chicago, IL, USA) to ascertain the frequency distribution of the blood groups. Descriptive statistics (frequency counts and percentages) was used to describe ABO/Rh(D) distribution under the study demographic characteristics (sex, age groups and ethnic groups). Descriptive analysis was performed and presented as frequency counts and percentages in tables or graph. Chi-square test was used to also test the association between distribution of blood groups and study demographic characteristics (sex, age groups and ethnic groups). P < 0.05 was considered statistically significant.

### Data stratification

All ethnic groups within the region were placed under four broad ethnicities - (Akans, Ewes, Ga-Adamgbes and Northerners) because of common ancestral routes, different indigenous socio-cultural beliefs and practices, language barriers and inter-ethnic marriage limitations. Hence, the data of the research is also broken accordingly. All other subjects of non-indigenous Ghanaian family routes were classified as non-Ghanaian.

## Results

Overall distribution of blood groups in Volta region (Table 1)

**Table 1:** Distribution of ABO/Rhesus factor blood groupings amongst sexes and the overall population in the Volta Region of Ghana

Characteristic	Blood group/Rhesus factor								Total freq. N (%)
Sex	A+ n (%)	A- n (%)	B+ n (%)	B- n (%)	AB+ n (%)	AB- n (%)	O+ n (%)	O- n (%)	
Male	1501 (20.1)	112 (1.5)	1699 (22.8)	90 (1.2)	204 (2.7)	6 (0.1)	3463 (46.4)	392 (5.2)	7467 (52.0)
Female	1213 (17.6)	130 (1.9)	1800 (26.1)	90 (1.3)	236 (3.4)	4 (0.1)	3180 (46.1)	240 (3.5)	6893 (48.0)
Overall (N%)	2714 (18.9)	242 (1.7)	3499 (24.4)	180 (1.3)	440 (3.1)	10 (0.1)	6643 (46.3)	632 (4.4)	**14360**

Pearson χ2 (7) test = 63.313. p < 0.001. Note: values in bracket denoted degree of freedom. Data is tabulated as observed frequencies with (percentages).

A total of 14,360 (52% males and 48% females) ABO/Rh blood group data on donors and recipients were retrieved. The blood group frequency among sexes and the overall population is presented in Table 1. The overall distribution of ABO/Rh blood groups was 46.30%, 18.91%, 24.41%, 3.11%, 4.41%, 1.71%, 1.31% and 0.10% for O+, A+, B+, AB+, O-, A-, B- and AB- blood groups respectively (Table 1). The frequency of blood group O+ was the highest with AB being the lowest within the study population. Rh (D)+ to Rh (D)- ratio was 92.5 to 7.5% respectively (Table 1).

### Distribution of blood groups based on ethnic affiliation (Table 2)

Among all the ethnic groups, blood group O+ was the highest (> 35%) whereas AB- blood group was the least (< 1%). The proportion of blood groups AB+, O-, A-, B- and AB- were relatively lower (ranged 0 – 6%) than blood groups O+, A+ and B+ (ranged 19 – 47%) in all ethnic groups. (Table 2).

**Table 2:** Distribution of ABO/Rhesus factor blood groupings amongst ethnic groups in the Volta Region of Ghana

Characteristic	Blood group/Rhesus factor								Total freq. N (%)
Ethnic group	A+ n (%)	A- n (%)	B+ n (%)	B- n (%)	AB+ n (%)	AB- n (%)	O+ n (%)	O- n (%)	
Ga Adamgbe	277 (20.9)	27 (2.0)	280 (21.1)	27 (2.0)	46 (3.5)	4 (0.3)	616 (46.4)	50 (3.8)	1327 (9.24)
Akan	46 (12.7)	3 (0.8)	118 (32.6)	1 (0.3)	13 (3.6)	1 (0.3)	167 (46.1)	13 (3.6)	362 (2.52)
Ewe	2378 (19.0)	204 (1.6)	3030 (24.2)	145 (1.2)	365 (2.9)	7 (0.1)	5797 (46.4)	580 (4.6)	12506 (87.09)
Northern	33 (32.4)	3 (2.9)	13 (12.7)	3 (2.9)	6 (5.9)	1 (1.0)	36 (35.3)	7 (6.9)	102 (0.71)
Non-Ghanaian	12 (19.0)	1 (1.6)	12 (19.0)	1 (1.6)	1 (1.6)	1 (1.6)	29 (46.0)	6 (9.5)	63 (0.44)

Pearson χ2 (28) test =104.170. p < 0.001. Note: values in bracket denoted degree of freedom. Data is tabulated as observed frequencies with (percentages).

### Distribution of blood groups based on age groups (Table 3)

**Table 3:** Distribution of ABO/Rhesus factor blood groupings amongst age groups in the Volta Region of Ghana

Characteristic	Blood group/Rhesus factor								Total freq. N (%)
Age group	A+ n (%)	A- n (%)	B+ n (%)	A- n (%)	AB+ n (%)	AB-n (%)	O+ n (%)	O-n (%)	
0–20	286 (14.6)	26 (1.3)	494 (25.3)	10 (0.5)	135 (6.9)	3 (0.2)	906 (46.3)	96 (4.9)	1956 (13.62)
21–40	2009 (19.7)	198 (1.9)	2358 (23.2)	142 (1.4)	234 (2.3)	7 (0.1)	4726 (46.4)	510 (5.0)	10184 (70.92)
41–60	408 (20.2)	9 (0.4)	497 (24.6)	26 (1.3)	59 (2.9)	3 (0.1)	971 (48.0)	49 (2.4)	2022 (14.08)
Above 60	42 (21.2)	3 (1.5)	46 (23.2)	1 (0.5)	3 (1.5)	1 (0.5)	101 (51.0)	1 (0.5)	198 (1.38)

Pearson χ2 (21) test = 220.854. p < 0.001. Note: values in bracket denoted degree of freedom. Data is tabulated as observed frequencies with (percentages).

The variations in the frequency distribution among different age groups (Table 3) followed a similar pattern. Blood group O+ was most predominant in all the age groups (> 46 %).

## Discussion

Availability of established information on blood groupings in any population is of great importance. For example, in blood transfusion services, information on blood groups of donors and recipients is necessary. This is because a donor's blood group must be compatible with that of the recipient; otherwise, agglutination of donor blood cells may occur in the plasma of the recipient^11^. Due to this clinical importance with regards to blood transfusion, obtaining information on the ABO/Rh blood group distribution for every population has been of significance for years. Furthermore, blood group frequency distribution pattern is reported to vary from race to race and among populations^11^.

The findings of this study on the ABO distribution are represented by a general formula O+/- > B+/- > A+/- > AB+/-, showing that blood group O is most predominant within the population and AB has the least frequency in the Volta region of Ghana. These findings are consistent with the trend reported in studies carried out in other regions in Ghana^5^,^10^ and in a Nigerian population^12^. This study, however, does not agree with some reported results for some African countries^1^,^2^,^13^ and some other parts of the world^14^, where the reported trend was O > A > B > AB. Trends in blood group A or B frequencies may be comparable or show minimal differences depending on the population in Africa^1^,^2^,^13^. Our findings, however, generally agreed with all available data that Africans are predominantly of blood group O, with AB group being the least represented^1^,^2^,^5^,^10^,^12^,^13^. Our findings are also in agreement with some Western populations although some studies have shown variations in other races. For instance, studies in India^15^ and Pakistan^16^ showed the trend B > O > A > AB. Again, the trend A > O > B > AB has also been reported in another study done in Nepal^17^. Blood group AB, regardless of the differences in the trends reported above, has been the group with the least distribution among the populations. From the study, we report that the Volta region of Ghana has an advantage in blood transfusion services since its prevalent blood group is the most compatible donor blood group and its readily available. The prevalence of blood group O as reported in this study is also seen in other available Ghanaian studies on frequency of blood groups for Greater Accra region^5^ and Eastern region^10^ and among selected populations^18^,^19^. This available evidence predicts that other parts of the country are likely to have blood group ‘O’ as the prevalent blood group. We highly recommend future studies to ascertain the frequency of blood groups in nationwide for effective blood transfusion services nationwide.

This study reports the predominance of Rh (D)+ (92.5%), with Rh (D)- (7.5%) having a relatively lower percentage frequency in the Volta region of Ghana. This observation is in agreement with the study reported for the Greater Accra and Eastern regions of Ghana^5^,^10^. Again, this study, compared with previous findings, further confirms the low occurrence of rhesus negativity in the African, Western and Asian populations^13^,^20^. For eample, the frequency of Rh-negatives ranges between 1–7%, 6%, 1%, 0.6–8.4% in Ethiopia, Nigeria, Madagascar and India respectively^13^. Studies in China, Indonesia and Japan also reported that the proportion of Rh-negative in the population is below 1%. Britain and the United States are also reported to have a proportion of 17 and 15% of their population being Rh factor negative, respectively^13^. A regional study in Saudi Arabia and among Basques of Morocco reported a 29% Rh-negative, which is the highest reported prevalence in any population^13^. Knowledge on Rhesus blood system is important to prevent erythroblastosis fetalis (haemolytic disease of the newborn), which occurs in a Rhesus-negative mother carrying Rhesus-positive fetus. Per our findings, we expect the number of cases of haemolytic disease of the newborn in the Volta region to be much lower because of the low percentage frequency of Rh-negative. Future studies could examine the prevalence of haemolytic disease of the newborn in the Volta region.

Our data suggests there is a chance to believe that ABO/Rhesus blood group distribution is associated with gender (p-value < 0.001). We also report a trend thus O+ > B+ >A+ > AB+ > O- > B- >A- > AB- for each gender as similarly reported by Doku et al., 5 and Kretchy et al.,^10^ in their studies for other regions of the country^5^,^10^. The slight proportional difference can be as a result of different sample sizes for males and females.

Ethnic group distribution was considered in this study because of emigrational influences on the native population. Although the region is dominated with the Ewe ethnic group (68.5 percent of the population), the current trend of societal migration has allowed for significant representation of other ethnicities in the region. Association between blood group distribution and ethnic groups showed a p-value < 0.001(highly significant) which indicates a reason to suggest there is a relationship between blood group distribution and blood group. ABO/Rh frequency distribution among the various ethnic groups had a comparable trend which is not different from observations made by Doku et al.,^5^ and Kretchy et al.,^10^ in their studies for other regions in Ghana. The study indicates blood group O as the most highly distributed blood group among the various ethnic groups in the Volta region of Ghana. Golassa et al.,^13^ reported that blood group O and A were most prevalent among the Nilotic natives and ‘highlanders’, respectively, within the population of Gambella Town in Ethiopia. These reports ABO frequency distribution can differ among different ethnic groups within a population. It is thus important to obtain established information on the frequency distribution among ethnic groups for any notable population for effective blood transfusion services^5^,^10^,^13^.

The results on age group distribution shows that, for decades to come, blood groups AB+, O-, A-, B- and ABwill be represented by relatively very small distributions, whilst O+, A+ and B+ will be the prevalent blood groups in the population. Our findings reported the trend O+ > B+ or A+> AB+ > O- > B- or A- > AB- among all age groups as observed by Doku et al^5^ and Kretchy et al.,^10^ for Greater Accra and Eastern regions of Ghana respectively. We also report that there is a likelihood that age groups have an association with blood group distribution (p-value < 0.001). Knowledge of blood group distribution by age is relevant, in that it enables for the prediction of the pattern of distribution within a population over the decades to come.

## Limitations of study

Retrospective studies are designed to analyze pre-existing data and are subject to numerous biases as a result. We were also limited in knowing the family relationships between donors and recipients. These limitations, notwithstanding, the study adds to the existing knowledge about prevailing blood group phenotype/Rhesus factor among the population and could influence decisions about blood banking in health facilities within the Volta region of Ghana.

## Conclusion

This study presents data on the ethnic distribution and ABO/Rh blood group frequency in a major administrative region of Ghana. The population of Volta region are seen to be dominant in blood group O as well as majority being Rh-positive. Data reported in this study population showed an ABO blood group distribution similar to the reported African trend. Future studies must also include serological and genetic investigation of the prevailing ABO blood group antigens/Rhesus factor in the population. The study findings are useful for health care planning, blood bank inventory, population genetic studies, genetic counseling, clinical investigations as well as the functional wellbeing of people in the population.
